# Supplementary material for: Bulk Photovoltaic Current Mechanisms in All-Inorganic Perovskite Multiferroic Materials
Source: Nanomaterials (Basel). 2023 Jan 20;13(3):429. doi: 10.3390/nano13030429 (PMC9920813; doi:10.3390/nano13030429)
Supplement: Supplementary file 1 [file nanomaterials-13-00429-s001.zip › nanomaterials-2167973-supplementary.pdf]

Supplementary Material

Bulk photovoltaic current mechanisms in all-inorganic perovskite multiferroic materials

Jiazheng Chen [1,†], Guobin Ma [1,2,†], Boxiang Gong [2], Chaoyong Deng[2], Min Zhang [1], Kaixin Guo [2], Ruirui Cui [1], Yunkai Wu [1], Menglan Lv [4] and Xu Wang[1, 3*]

[1]Key Laboratory of Functional Composite Materials of Guizhou Province,
College of Big Data and Information Engineering, Guizhou University, Guizhou 550025, China

[2]School of Electronics and Information Engineering, Guiyang University, Guiyang 550005, China

[3]Guiyang Makers Center, Guizhou 550025, China

[4]School of Chemistry and Chemical Engineering, Guizhou University, Guizhou 550025, China

Corresponding Authors

*E-mail: xuwang@gzu.edu.cn

A. log (J) -log (E) curves to illustrate leakage mechanism

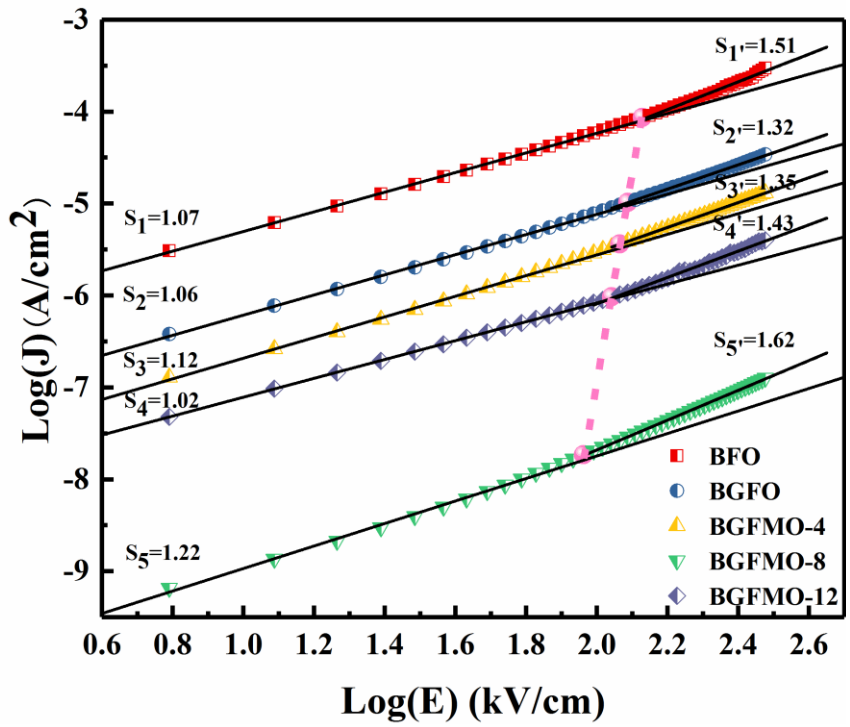

Figure S1. The log (J) -log (E) curves of different samples.

To understand the leakage mechanism better, the log (J) -log (E) curve was drawn (shown in Figure S1.). The curve exhibits an excellent linearity (i.e. J∝E, where approaches 1), The leakage conduction mechanism of the films is manifested as an ohmic contact. The conduction formula is shown as follows,

$$J = e\mu NeE$$

where e, μ, Ne and E are the charge of electrons, the carrier mobility of free electrons, the density of free electrons, and the applied field, representively. In the course of the experiment, the generated oxygen vacancies caused by the volatilization of Bi formed a deep-well energy level in the bandgap so that the activated electrons can move freely. Therefore, the more oxygen vacancies, the more free carriers will be generated in the film. One can identify the transition point indicated by the doted pink line, which corresponds the separation and merging upon Gd and Mn co-doping as shown in the inset of Figure 1 (a).

B. Amplified Linear and circular leakage current I-V results

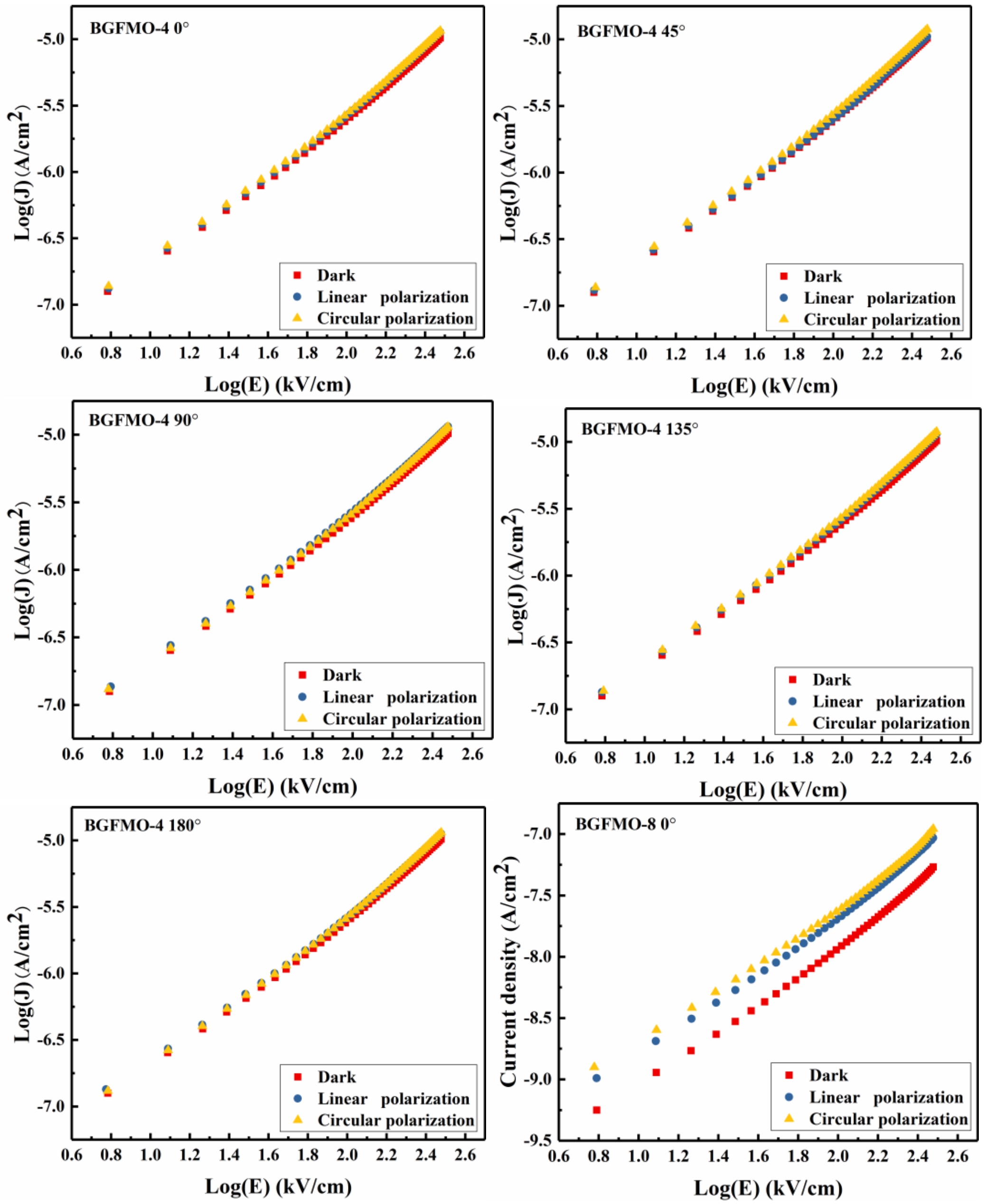

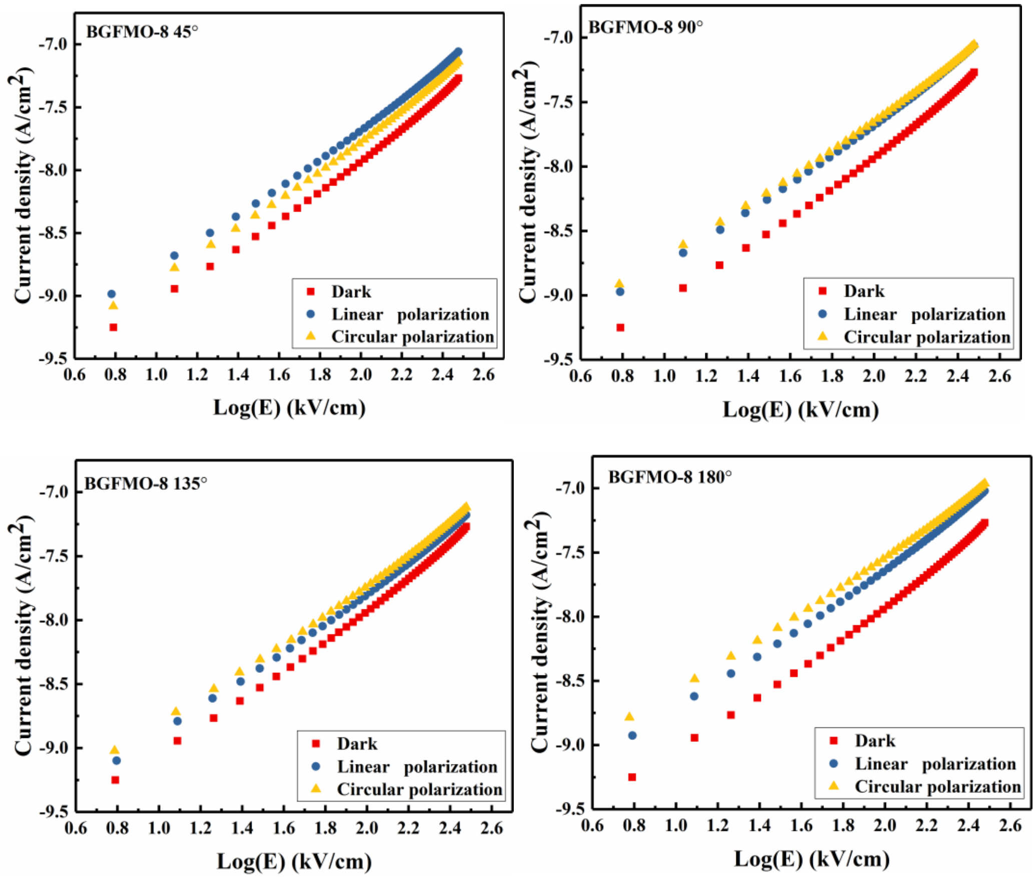

Figure S2. The log (J) -log (E) curves of circular polarization and linear polarization of BGFMO-4 and BGFMO-8 at different rotation angles under polarization-modulated commercial 405 nm laser illumination.

C. The trend of leakage current I-V results

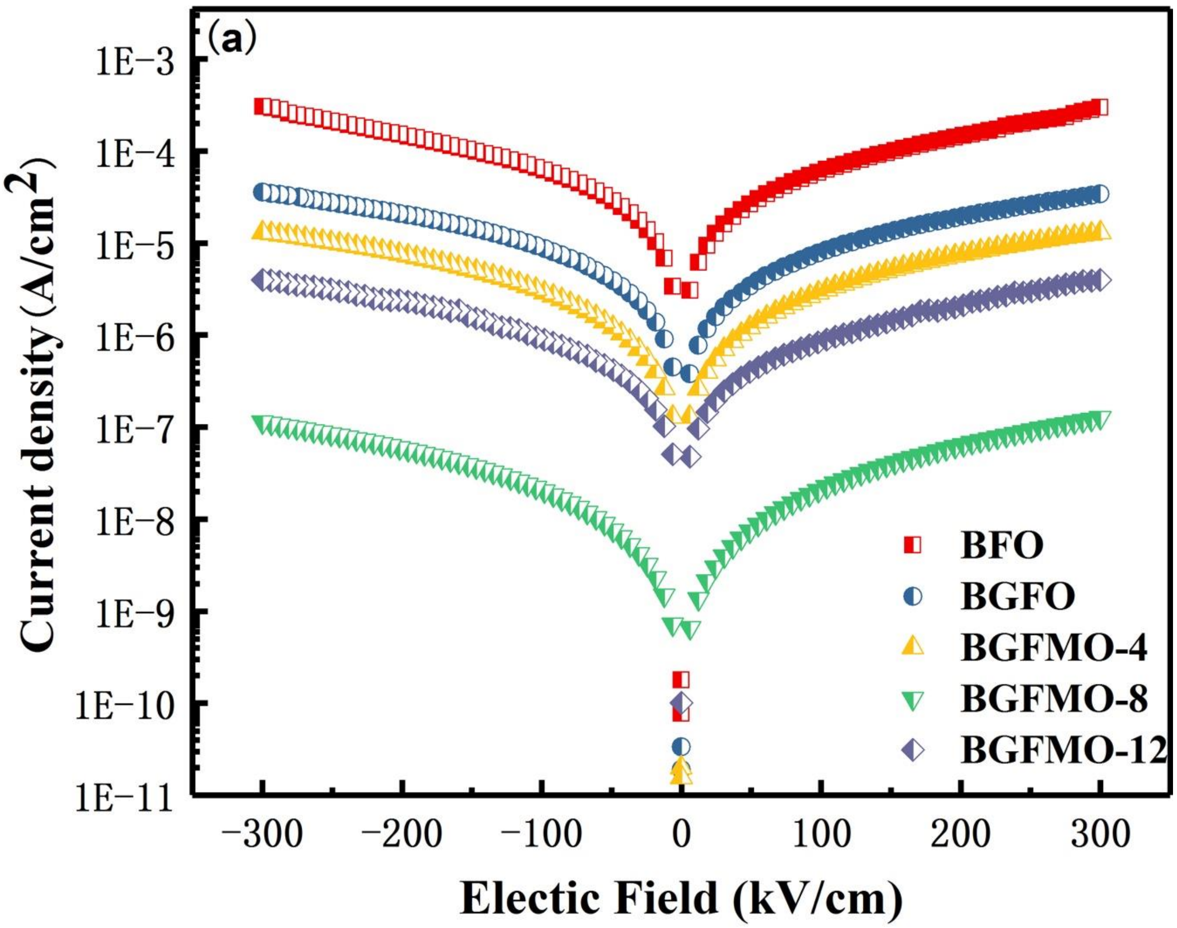

Figure S3 The leakage current density (J-E) curves of each doping concentration measured at room temperature.

Fig. S3 shows the leakage current density (J-E) curves of each doping concentration measured at room temperature. All the J-E curves exhibit excellent symmetrical structure. Gd, Mn co-doping results in reducing the leakage current density from 5.7×10^{-2} A/cm^2 for BFO to 7.39×10^{-8}A/cm^2 at 6 V when the Mn doping concentration is 8%. One reason for this is that the co-doping remarkably reduces the size of grains (from over 150 nm to ～20 nm) so as to yield an increase in the density of grain boundary, which benefits to charge separation and enhance the photo generated carrier conductivity. To understand the leakage mechanism better, the log (J) - log (E) curve shown in Fig. S1 exhibited an excellent linearity (with a transition point of each curve corresponding to the structural transformation mentioned in the XRD rusults), which manifested the leakage conduction mechanism of the films with the Ohmic conduction contacts. The Ohmic mechanism indicates the significant influence of resistivity brought by morphologies, so as to share the similar trend of discussed in the paper.
